# Early CD4^+^ T cell responses induced by the BNT162b2 SARS-CoV-2 mRNA vaccine predict immunological memory

**DOI:** 10.1038/s41598-022-24938-4

**Published:** 2022-11-27

**Authors:** Jie Bai, Asako Chiba, Goh Murayama, Taiga Kuga, Yoshiyuki Yahagi, Yoko Tabe, Naoto Tamura, Sachiko Miyake

**Affiliations:** 1 Department of Immunology, Juntendo University Graduate School of Medicine, Tokyo, 113-8421 Japan; 2 Department of Internal Medicine and Rheumatology, Juntendo University School of Medicine, Tokyo, 113-8421 Japan; 3 Department of Clinical Laboratory Medicine, Juntendo University Graduate School of Medicine, Tokyo, 113-8421 Japan

**Keywords:** Cellular immunity, Humoral immunity, RNA vaccines

## Abstract

Longitudinal studies have revealed large interindividual differences in antibody responses induced by SARS-CoV-2 mRNA vaccines. Thus, we performed a comprehensive analysis of adaptive immune responses induced by three doses of the BNT162b2 SARS-CoV-2 mRNA vaccines. The responses of spike-specific CD4^+^ T cells, CD8^+^ T cells and serum IgG, and the serum neutralization capacities induced by the two vaccines declined 6 months later. The 3^rd^ dose increased serum spike IgG and neutralizing capacities against the wild-type and Omicron spikes to higher levels than the 2^nd^ dose, and this was supported by memory B cell responses, which gradually increased after the 2^nd^ dose and were further enhanced by the 3^rd^ dose. The 3^rd^ dose moderately increased the frequencies of spike-specific CD4^+^ T cells, but the frequencies of spike-specific CD8^+^ T cells remained unchanged. T cells reactive against the Omicron spike were 1.3-fold fewer than those against the wild-type spike. The early responsiveness of spike-specific CD4^+^ T, circulating T follicular helper cells and circulating T peripheral helper cells correlated with memory B cell responses to the booster vaccination, and early spike-specific CD4^+^ T cell responses were also associated with spike-specific CD8^+^ T cell responses. These findings highlight the importance of evaluating cellular responses to optimize future vaccine strategies.

## Introduction

mRNA vaccines that express the spike glycoprotein of severe acute respiratory syndrome coronavirus 2 (SARS-CoV-2) have been used worldwide and have been shown to be effective in controlling the pandemic of coronavirus disease 2019 (COVID-19)^1–8^. However, the effectiveness of mRNA vaccines appears to wane over time, as vaccine efficacy against SARS-CoV-2 infection has been shown to decrease approximately 20–30% 6 months after vaccination^4,8–10^. Serum antibody levels decreased 6–8 months after induction by two doses of the SARS-CoV-2 mRNA vaccine, but a 3rd dose induced a robust increase in antibody levels, which were even higher than those after the 2nd dose. The 3rd dose effectively increased the levels of neutralizing antibodies against not only the Wuhan strain but also variant strains of SARS-CoV-2, such as Delta (B.1.617.2) and Omicron BA.1 (B.1.1.529)^4,11–13^. However, longitudinal studies on SARS-CoV-2 mRNA vaccine-induced antibody responses in healthy adults revealed large interindividual differences in serum antibody levels^14–17^. Some individuals maintained high levels of antibodies 6–8 months after the 2nd dose, sometimes even at levels higher than those seen in the peak responses in some individuals after the 2nd or 3rd dose. Some people also showed very weak antibody responses after the 2nd dose, and thus the serum levels became low within a few months. Therefore, although a fourth dose of the SARS-CoV-2 mRNA vaccine is currently recommended for immunocompromised individuals, some healthy individuals may also need a booster vaccine to maintain antiviral immunity.

Antiviral vaccine efficacy has been mostly discussed in the context of antibody responses. Indeed, many studies on immune responses following COVID-19 vaccination have focused on antibody responses against the SARS-CoV-2 spike. However, cellular responses specific to the spike are important for antiviral immunity against SARS-CoV-2^18–20^. Spike-specific memory B cells are thought to produce anti-spike antibodies when they encounter the virus. SARS-CoV spike-specific CD8^+^ cytotoxic T cells have been shown to destroy cells infected with SARS-CoV^21–23^. CD8^+^ T cell activation requires CD4^+^ helper T cells that recognize the same antigen and activate antigen-presenting cells. CD4^+^ helper T cell subsets known as T follicular helper (Tfh) and T peripheral helper (Tph) cells provide cognate help to B cells become antibody-producing cells and memory B cells. In COVID-19, higher responses of CD8^+^ T cells and CD4^+^ T cells, including circulating Tfh (cTfh) cells, against SARS-CoV-2 are associated with milder disease^24–26^. The associated responses of cTfh cells and antibodies against SARS-CoV-2 also correlate with reduced disease severity^27–31^. Clonally expanded CD8^+^ T cells in bronchoalveolar lavage fluid were observed in patients with moderate disease^32^. The depletion of CD8^+^ T cells has been shown to partially abrogate the protection against rechallenge with SARS-CoV-2 in convalescent rhesus macaques^33^. Thus, a comprehensive analysis of the adaptive immune system, including cellular components, is desired to evaluate antiviral immunity against SARS-CoV-2.

Many studies, including ours, have shown that two doses of SARS-CoV-2 mRNA vaccines induced the spike-specific responses of CD8^+^ T and CD4^+^ T cells, including cTfh and circulating Tph (cTph) cells^34–42^. The spike-specific T cells waned over time, and 6 months later, while spike-reactive CD4^+^ T cells were present in most individuals, spike-reactive CD8^+^ T cells were observed among only half of the individuals. Some studies have shown that the 3rd dose of SARS-CoV-2 mRNA vaccine induced higher T cell responses^43,44^, but other studies have demonstrated that T cell responsiveness was unchanged by the 3rd dose^45–48^. In contrast to the effect on antibody responses, the 3rd dose did not increase T cell epitopes and did not enhance T cell responses against the omicron variant^49–51^. Because T cell responses against the SARS-CoV-2 spike have been evaluated using different methods, such as enzyme-linked immunospot assays for cytokine production and activation-induced marker assays using fresh or freeze-thawed peripheral blood mononuclear cells, the magnitude and maintenance of T cell responses elicited by the 3rd dose remain unknown.

Peripheral blood mononuclear cells (PBMCs) and serum were collected before and 1 week, 2 months and 6 months after the 2nd dose of the mRNA vaccine and 2 months after the 3rd dose, and we analyzed the spike-specific responses of antibodies, CD4^+^ T cells, CD8^+^ T cells, and B cells with regard to their persistence and responsiveness to the booster vaccine. Two doses of the mRNA vaccine induced the spike-specific responses of the antibodies, CD4^+^ T cells, and CD8^+^ T cells in all participants of the study. However, the persistence after the 2nd dose and the responsiveness to the booster dose were different among these components of adaptive immunity. The serum antibody levels and neutralizing capacities showed large decreases 6 months later, but the 3rd dose of immunization induced antibody responses, including those against the Omicron variant, to even higher levels than the 2nd dose did. Interestingly, the levels of spike-specific memory B cells gradually increased after the 2nd dose and increased further after the 3rd dose. The reduction in spike-specific T cell responses after the 2nd dose was relatively small, and the response of these T cells to the 3rd dose was minimal. T cell and antibody responses against the Omicron spike were reduced compared to those against the wild-type spike, but the reduction in responsiveness was much smaller in T cells than in antibodies. The responses of spike-specific CD4^+^ T cells and cTfh and cTph cells at 1 week after the 2nd dose were positively correlated with those of memory B cells after the 3rd dose, and early spike-CD4^+^ T cell responses were positively correlated with spike CD8^+^ T cell responses at all time points. These findings shed light on the differences among adaptive immune components and the importance of early CD4^+^ T cell responses that predict the memory of B and CD8^+^ T cells.

## Results

### Spike-reactive CD4^+^ T and CD8^+^ T cells were maintained after the two doses of vaccines, but they responded to the booster vaccine differently

**Figure 1:**
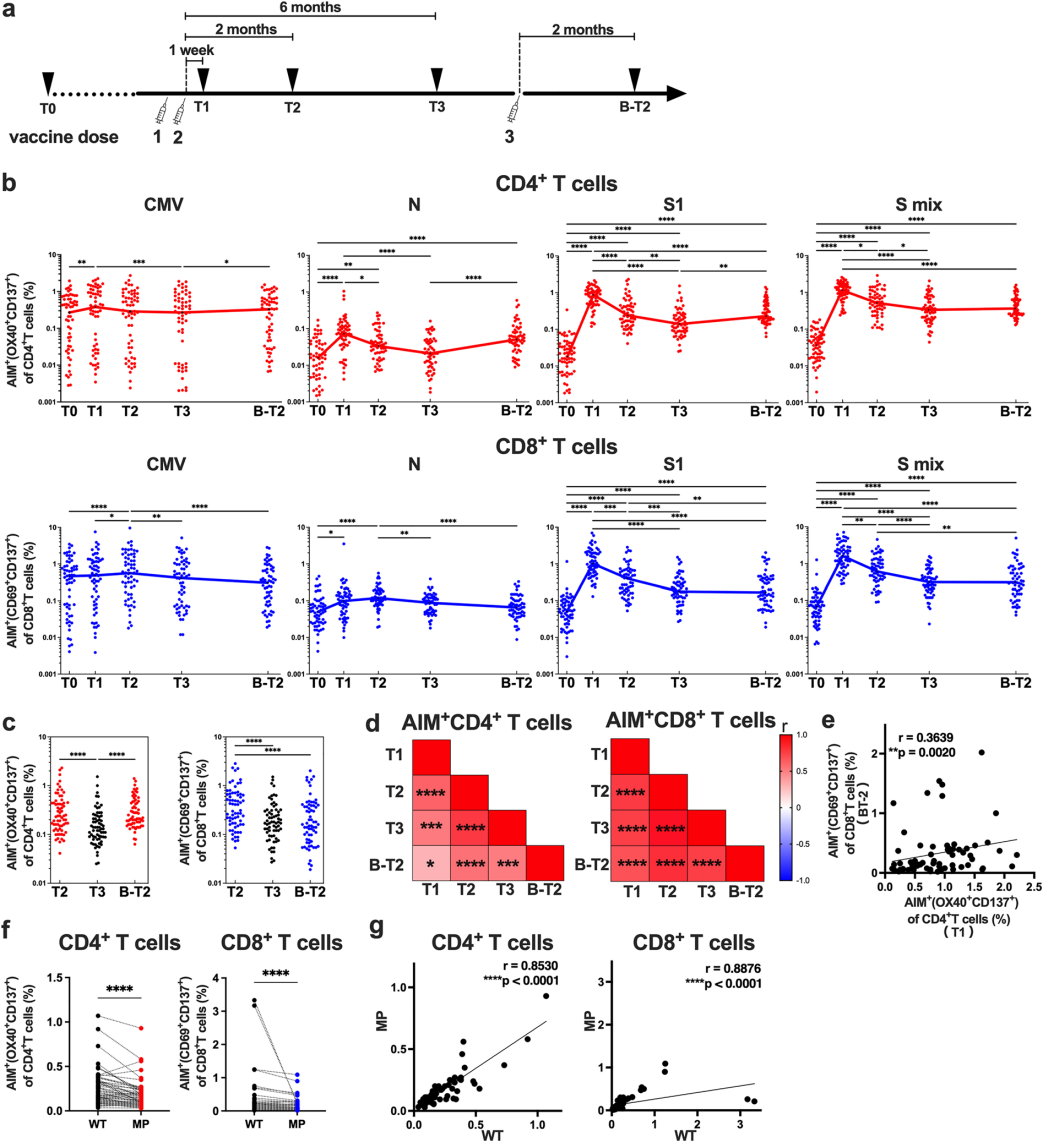
SARS-CoV-2 mRNA vaccine induced memory T cells against wild-type and Omicron spike proteins. Peripheral blood samples were obtained before (T0), 1 week (T1), 2 months (T2), and 6 months (T3) after the 2nd dose of the BNT162b2 mRNA vaccine and 2 months after the 3rd dose (B-T2). (**a**) The timeline of vaccinations and sample collections is shown. (**b**–**g**) Activation-induced marker (AIM) assays were performed using peripheral blood mononuclear cells (PBMCs) obtained at the indicated time points. PBMCs were stimulated with peptide pools of SARS-CoV-2 structural proteins, including the spike and nucleocapsid, cytomegalovirus pp65 protein as a control (**b**–**e**), and a mutant pool of the spike protein of the SARS-CoV-2 Omicron variant (MP) and wild-type reference pool (WT) (**f**, **g**). Twenty-two hours later, T cells responding to the indicated peptide pools were identified as AIM^+^ (OX40^+^ CD137^+^) CD4^+^ T cells and AIM^+^ (CD69^+^ CD137^+^) CD8^+^ T cells by flow cytometry. Frequencies of AIM^+^ CD4^+^ T and AIM^+^ CD8^+^ T cells at each time point (**b**) and at T2, T3, and B-T2 (**c**) are shown. (**d**) Heatmaps show the correlations among S1-reactive AIM^+^ CD4^+^ T cell frequencies (left) and S1-reactive AIM^+^ CD8^+^ T cell frequencies (right) at the indicated time points. (**e**) Correlation between the frequencies of S1-reactive AIM^+^ CD4^+^ T cells at T1 and S1-reactive AIM^+^ CD8^+^ T cells at B-T2. (**f**) Frequencies of AIM^+^ CD4^+^ T and AIM^+^ CD8^+^ T cells reactive to MP and WT pools at B-T2. (**g**) Correlations between MP- and WT-reactive AIM^+^ CD4^+^ T cells and AIM^+^ CD8^+^ T cells at B-T2. CMV, cytomegalovirus pp 65 protein; N, SARS-CoV-2 nucleocapsid; S1, SARS-CoV-2 spike S1 domain; S mix, a mixture of SARS-CoV-2 spike S1 and S2 domains. Each dot indicates the value for one individual. Significant differences were analyzed by the Friedman test (**b**, **c**) and Wilcoxon matched-pairs signed-rank test (**f**). Correlations were analyzed using Spearman’s correlation analysis (**e**, **g**). *P < 0.05, **P < 0.01, ***P < 0.001, ****P < 0.0001.

To identify SARS-CoV-2 spike-reactive CD4^+^ T and CD8^+^ T cells, flow cytometry-based activation induced marker (AIM) assays were performed using PBMCs obtained before and 1 week, 2 months and 6 months after the 2nd dose of the mRNA vaccine and 2 months after the 3rd dose (Fig. 1a). PBMCs were stimulated with peptide pools of SARS-CoV-2 structural proteins, and antigen-specific AIM^+^ CD4^+^ T and AIM^+^ CD8^+^ T cells were identified as cells positive for “OX40^+^ CD137^+^” and “CD69^+^ CD137^+^” cells, respectively (Supplementary Fig. 1a). The peptide pools of SARS-CoV-2 used for AIM assays included “S1” (the complete N-terminal S1 domain of the spike glycoprotein including RBD; aa 1–692), “S mix” (mixed peptide pools of S1 and S2 domains) and “N” (the complete sequence of the nucleocapsid phosphoprotein). The peptide pool of the cytomegalovirus pp65 protein (CMV) was also used as a control. As previously reported by our group and other groups, S1- and S mix-reactive AIM^+^ CD4^+^ T cells and AIM^+^ CD8^+^ T cells were induced by the two doses of the mRNA vaccine^34,37,52–56^. The median percentages (interquartile [IQR]) of S1-reactive AIM^+^ CD4^+^ T cells and AIM^+^ CD8^+^ T cells at 1 week after the 2nd dose were 0.88% (0.48–1.15) and 1.04% (0.57–2.05), respectively (Fig. 1b). Although the percentages of these AIM^+^ T cells decreased over time, S1-reactive AIM^+^ CD4^+^ T and AIM^+^ CD8^+^ T cells were observed at 6 months after the 2nd dose of the mRNA vaccine (0.14% [0.09–0.25] and 0.18% [0.12–0.32], respectively). The 3rd dose of the mRNA vaccine increased the percentage of S1-reactive AIM^+^ CD4^+^ T cells to 0.23% (0.18–0.48), but the percentage of S1-reactive AIM^+^ CD8^+^ T cells remained unchanged (Fig. 1b). When we compared the frequencies of S1-reactive AIM^+^ T cells at 2 months after the 2nd and 3rd doses, there was no significant difference in the percentages of these AIM^+^CD4^+^ T cells, but the percentage of AIM^+^ CD8^+^ T cells after the 3rd dose vaccine (0.17% [0.09–0.41]) was lower than that after the 2nd dose (0.41% [0.17–0.67]) (Fig. 1c). Thus, the spike-reactive CD4^+^ T and CD8^+^ T cells induced by the SARS-CoV-2 mRNA vaccine were maintained for at least 6 months, and the responsiveness to the booster vaccine was different between CD4^+^ T and CD8^+^ T cells. The frequencies of S1-reactive AIM^+^ CD4^+^ T cells at different time points were positively correlated, as were those of S1-reactive AIM^+^ CD8^+^ T cells (Fig. 1d). Interestingly, the frequencies of S1-reactive AIM^+^ CD4^+^ T cells at 1 week after the 2nd dose and AIM^+^CD8^+^ T cells at 2 months after the 3rd dose were positively correlated (Fig. 1e). The frequencies of S1-reactive CD4^+^ T and CD8^+^ T cells at each time point were also positively correlated with each other at all time points (Supplementary Fig. 1b).

We also investigated the T cell responses against the spike of the Omicron BA.1 variant (B1.1.529). PBMCs at 2 months after the 3rd dose were stimulated with a spike peptide pool of the Omicron BA.1 variant and a reference wild-type peptide pool, and AIM assays were performed. We observed that CD4^+^ T and CD8^+^ T cells responded to the Omicron peptide pool, but the frequencies of both AIM^+^ CD4^+^ T and AIM^+^ CD8^+^ T cells responding to the Omicron peptide pool showed a 1.3-fold reduction compared to those of the wild-type pool (Fig. 1f). The frequencies of AIM^+^ CD4^+^ T and CD8^+^ T cells reactive to the wild-type and Omicron spike peptide pools were positively correlated (Fig. 1g).

### The spike-specific antibody responses after the 2nd and 3rd doses were more dramatic than the T cell responses

**Figure 2:**
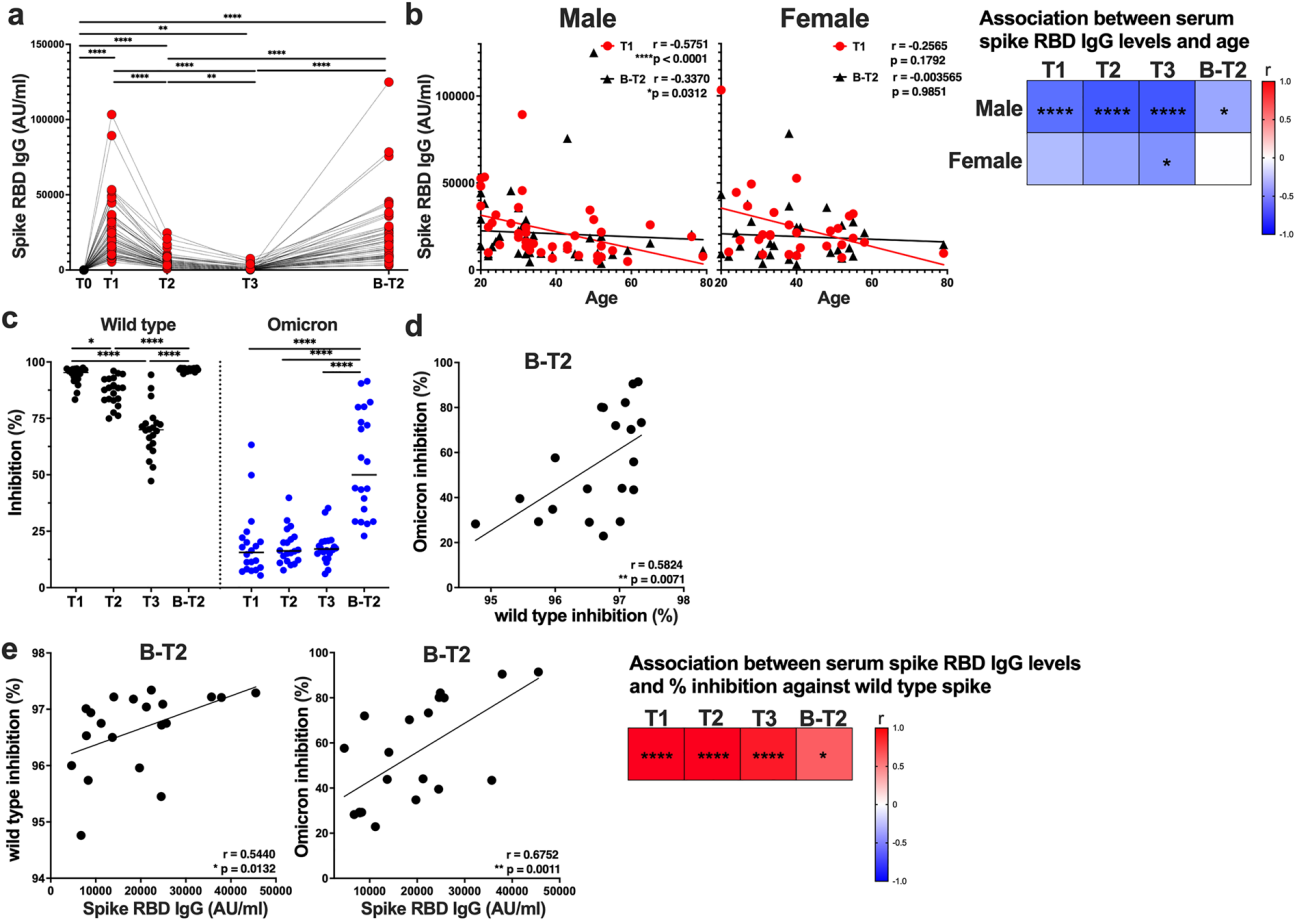
SARS-CoV-2 mRNA vaccine induced serum antibody titers and neutralizing activity. Serum samples were obtained before (T0) and 1 week (T1), 2 months (T2), and 6 months (T3) after the 2nd dose of the BNT162b2 mRNA vaccine and 2 months after the 3rd dose of the vaccine (B-T2). (**a**) Serum concentrations of the SARS-CoV-2 spike receptor binding domain (RBD) IgG at the indicated time points. (**b**) Graphs showing correlations between age and serum spike RBD IgG levels at T1 and B-T2 in males and females. Heatmap showing the associations of age with serum spike RBD levels at T1, T2, T3, and B-T2 in males and females. (**c**) Capacities of the sera to inhibit the interaction of angiotensin-converting enzyme 2 receptor and spike RBD proteins of wild-type (WT) SARS-CoV-2 and the Omicron variant. (**d**) Correlation between serum inhibiting capacities against the WT-RBD and Omicron-RBD at B-T2. (**e**) Graphs showing the correlation between serum spike RBD IgG levels and inhibition capacity against WT-RBD (left) and Omicron-RBD (right) at B-T2. Heatmap showing the correlations between serum spike RBD IgG levels and neutralizing capacities at T1, T2, T3, and B-T2. Each dot indicates the value for one individual. Significant differences between groups were analyzed by the Friedman test. Correlations were analyzed using Spearman’s correlation analysis. *P < 0.05, **P < 0.01, ***P < 0.001, ****P < 0.0001.

Next, we assessed the antibody response against the SARS-CoV-2 spike RBD IgG induced by the SARS-CoV-2 mRNA vaccine. The median serum concentration of spike RBD IgG at 1 week after the 2nd dose was 18716 AU/mL (11240-31947) and gradually decreased at 2 months (3866 AU/mL [2325-5865]) and at 6 months (923.3 AU/mL [600.4–1603]) after the 2nd dose, and the 3rd dose increased the serum spike RBD IgG levels to 15318 AU/mL (9065-25278) (Fig. 2a). Previously, we reported that the serum spike RBD IgG levels at 1 week after the 2nd dose were negatively correlated with age in males but not in females. Negative correlations between the serum spike RBD IgG levels and age were observed at all time points after the 2nd dose in males but only at 6 months after the 2nd dose in females (Fig. 2b). The negative correlation of the serum spike RBD IgG levels with age in males observed after the 3rd dose was weaker than that after the 2nd dose (Fig. 2b). We also investigated the neutralizing capacity of sera elicited by the mRNA vaccine. The sera were tested for their capacity to inhibit the interaction of angiotensin-converting enzyme 2 receptor and the SARS-CoV-2 wild-type or Omicron spike proteins. The median percentage of inhibition against the wild-type spike was 95.3% (92.3–96.8) at 1 week after the 2nd dose and decreased at 2 months (88% [83–92.5]) and at 6 months (69.9% [62.7–72.9]) after the 2nd dose but increased after the 3rd dose to 96.9% (96.1–97.2) (Fig. 2c). As previously reported^15,17^, the inhibitory capacity of serum against the Omicron spike was low after the 2nd dose but was dramatically increased after the 3rd dose (Fig. 2c). The inhibitory capacities against the wild-type and Omicron spikes after the 3rd dose were positively correlated (Fig. 2d). As expected, there was a positive correlation between the SARS-CoV-2 IgG concentrations and the neutralizing capacity against the wild-type spike (Fig. 2e). The serum anti-spike RBD IgG levels were also associated with the neutralizing capacity against the Omicron spike after the 3rd dose (Fig. 2e). Taken together, the results showed that 3rd dose of the mRNA vaccine provides benefits by enhancing humoral immunity against the Omicron variant and overcoming the disadvantages of reduced antibody responses with increasing age in males.

### Spike-specific memory B cells were gradually increased in response to the two vaccine doses and were further expanded by the 3rd dose

**Figure 3:**
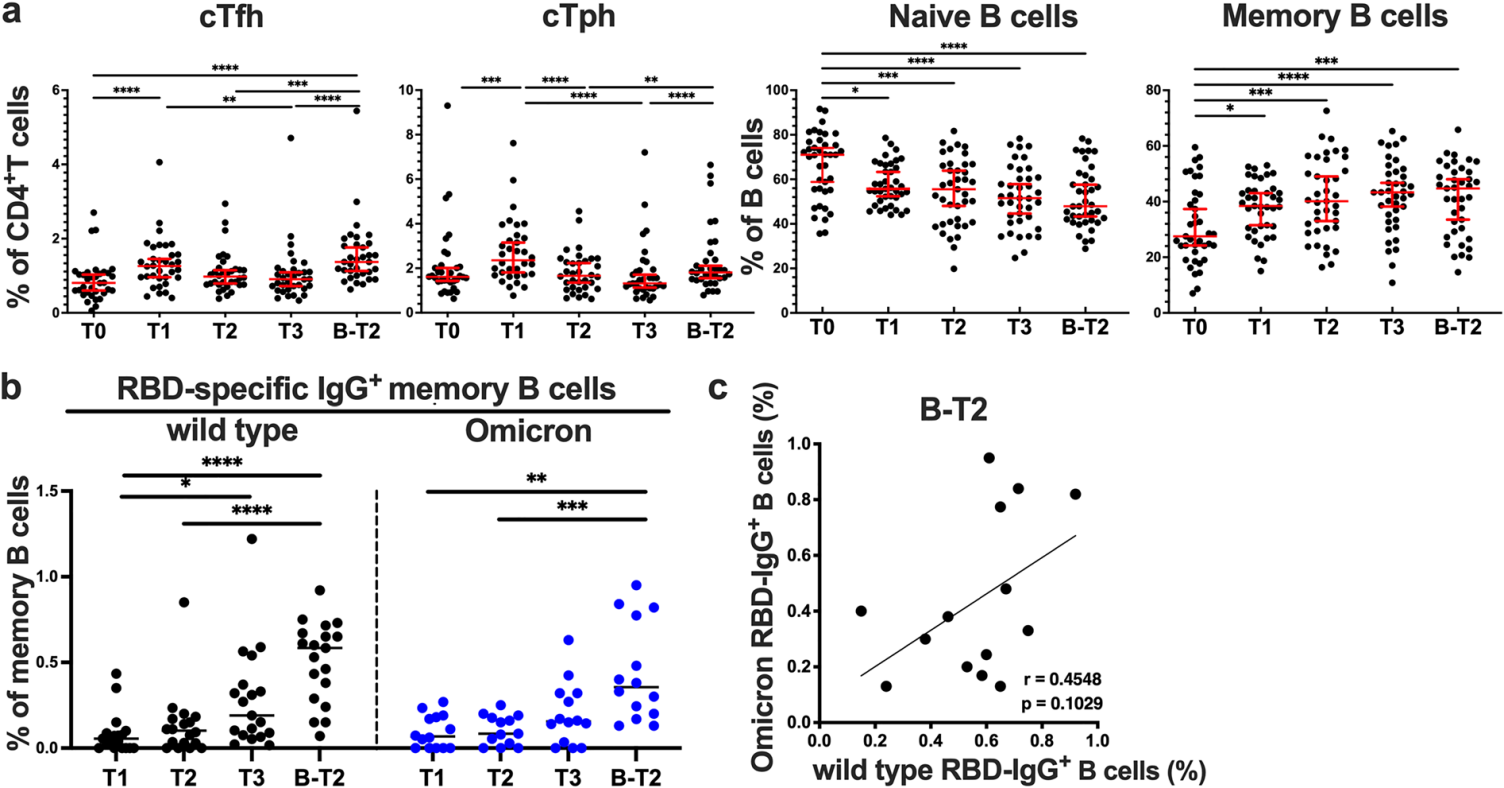
SARS-CoV-2 mRNA vaccine induced memory B cell responses. (**a**) The frequencies of circulating T follicular helper (cTfh) and circulating T peripheral helper (cTph) cells and naïve and memory B cells of peripheral blood mononuclear cells before (T0) and 1 week (T1), 2 months (T2), and 6 months (T3) after the 2nd dose of the BNT162b2 mRNA vaccine and 2 months after the 3rd dose of the vaccine (B-T2) are shown. (**b**) IgG^+^ memory B cells specific for the spike receptor-binding domain (RBD) of SARS-CoV-2 wild type and the Omicron variant induced by the BNT162b2 mRNA vaccine were analyzed by flow cytometry. The frequencies of wild-type and Omicron spike RBD-specific IgG^+^ cells among memory B cells at the indicated time points and (**c**) the association between these frequencies of wild-type and Omicron spike RBD-specific IgG^+^ at B-T2 are shown. Each dot indicates the value for one individual. Significant differences between groups were analyzed by the Friedman test. Correlations were analyzed using Spearman’s correlation analysis. *P < 0.05, **P < 0.01, ***P < 0.001, ****P < 0.0001.

B cells recognize antigens via their B cell receptors and produce antibodies against the same antigen. The maturation of B cells to antigen-secreting plasma cells and memory B cells requires cognate help by Tfh and Tph cells that recognize the same antigens. Thus, we analyzed helper T and B cell subsets important for antibody responses by flow cytometry (Supplementary Fig. 2a,b). The levels of cTfh and cTph cells induced by the two doses of the SARS-CoV-2 mRNA vaccine were decreased at 6 months after the 2nd vaccine dose and were increased by the 3rd dose (Fig. 3a). The frequencies of memory B cells gradually increased at 1 week, 2 and 6 months after the 2nd dose, and the 3rd dose of the mRNA vaccine also tended to increase the levels of these B cells (Fig. 3a). Conversely, the frequencies of naïve B cells decreased after the vaccination (Fig. 3a).

We also assessed the B cell response specific for the SARS-CoV-2 spike. The spike RBD-specific IgG^+^ memory B cells were identified by flow cytometry as previously described (Supplementary Fig. 2c)^17,52,57^. The median frequencies of these B cells at 1 week, 2 months, and 6 months after the 2nd dose of the vaccine were 0.049% (0–0.090), 0.102% (0.0218–0.173), and 0.17% (0.074–0.413), respectively. Interestingly, IgG^+^ memory B cells specific for the wild-type RBD were gradually increased after the 2nd dose, and the percentages of these cells were higher at 6 months than at 1 week after the second dose. The frequencies of spike RBD-specific IgG^+^ memory B cells after the 3rd dose were higher than those at 1 week and 2 months after the 2nd dose (Fig. 3b). The 3rd dose further increased spike RBD-specific IgG^+^ memory B cells, and the frequencies of these B cells at 2 months after the 3rd dose were found to be higher than those at 6 months after the 2nd dose upon comparison between these two time points (p=0.0012 by Wilcoxon matched-pairs signed-rank test). The frequencies of IgG^+^ memory B cells specific for the Omicron spike RBD tended to increase at 6 months after the 2nd dose and were higher at 2 months after the 3rd dose compared to those at 1 week and 2 months after the second dose (Fig. 3b). Again, the frequencies of these cells after the 3rd dose were found to be higher than those at 6 months after the 2nd dose upon comparison between these two time points (*P* = 0.0002 by Wilcoxon matched-pairs signed-rank test). The percentages of IgG^+^ memory B cells specific to the wild-type and Omicron spike RBD tended to be positively correlated after the 3rd dose (Fig. 3c).

### Early CD4^+^ T cell responses elicited by the mRNA vaccine predict immunological memory responses

**Figure 4:**
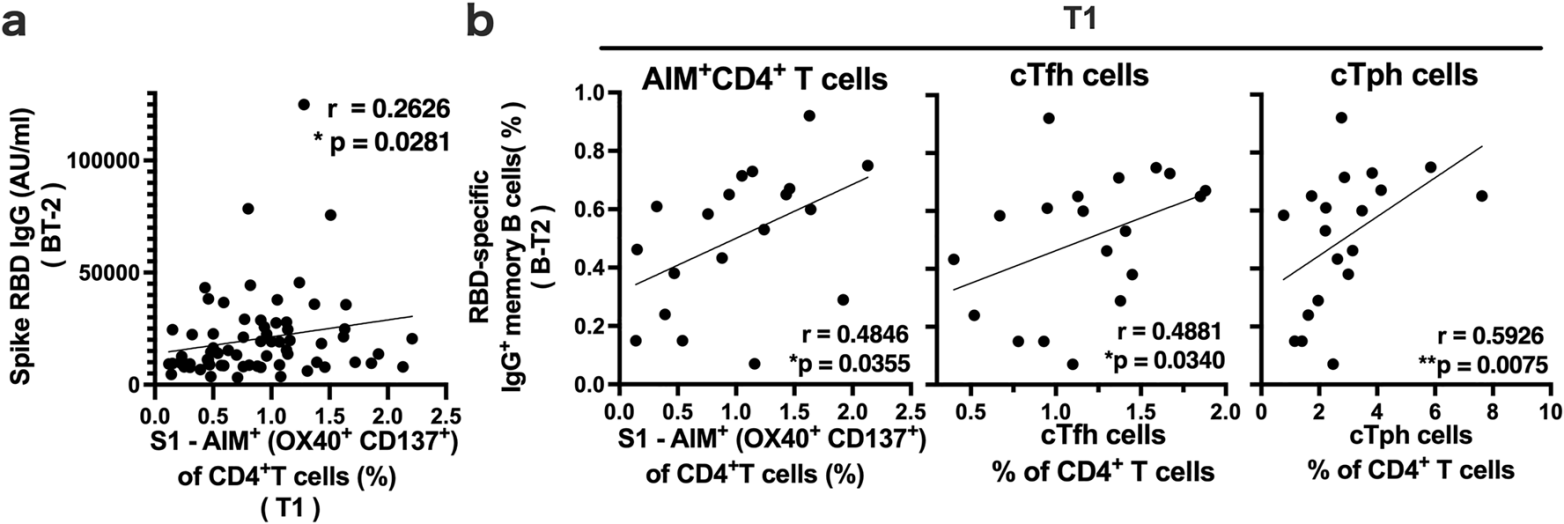
Association of SARS-CoV-2 mRNA vaccine-induced early CD4^+^ T cell responses and B cell memory. (**a**) The correlation between the frequencies of S1-reactive AIM^+^ CD4^+^ T cells at 1 week after the 2nd dose of the BNT162b2 mRNA vaccine (T1) and serum SARS-CoV-2 IgG levels at 2 months after the 3rd vaccine dose (B-T2) is shown. (**b**) Correlations of the frequency of SARS-CoV-2 spike RBD-specific IgG^+^ memory B cells at B-T2 with the frequencies of S1-reactive AIM^+^ CD4^+^ T cells, cTfh cells and cTph cells at T1 are shown. Each dot indicates the value for one individual. Correlations were analyzed using Spearman’s correlation analysis. *P < 0.05, **P < 0.01.

Because we observed large interindividual differences in the antibody responses elicited by the booster vaccine, we next asked whether antibody responses are associated with other adaptive immune responses (Supplementary Fig. 3a, b). The serum spike RBD IgG levels at 2 months after the 3rd dose were positively correlated with the frequencies of S1-specific AIM^+^ CD4^+^ T cells at 1 week after the 2nd dose, suggesting that the early CD4^+^ T cell response may impact the recall response of B cells (Fig. 4a, Supplementary Fig. 3a). The frequencies of spike-AIM^+^ CD4^+^ T cells at 1 week after the 2nd dose also showed a positive correlation with spike RBD-IgG^+^ memory B-cell frequencies at 2 months after the 3rd dose. The frequencies of spike RBD-IgG^+^ memory B cells after the 3rd dose were also positively correlated with those of cTfh and cTph cells 1 week after the 2nd dose (Fig. 4b, Supplementary Fig. 3b). These results indicate that early CD4^+^ T responses were associated with memory B cell responses to the booster vaccine.

## Discussion

We performed a comprehensive analysis of adaptive immune responses induced by the BNT162b2 SARS-CoV-2 mRNA vaccines and demonstrated that each component of the adaptive immune system responds in a different manner. Even though the antibody responses decreased over time after the two doses of vaccines, SARS-CoV-2 spike-specific CD4^+^ T and CD8^+^ T cells were maintained, and spike-specific memory B cells were even increased. The response to the booster vaccine also varied among antibodies and lymphocytes. These findings highlight the unique features of each player in the adaptive immune system and the importance of interrogating antigen-specific lymphocyte responses to evaluate the immunological memory elicited by the vaccines.

Circulating antibodies are thought to be produced by plasmablasts that respond rapidly to antigens^58,59^. It has been shown that the level of circulating spike reactive plasmablasts peaked one week after the 2nd BNT162b2 vaccine^52,60–62^. Previously, we also demonstrated that activated plasmablasts, which were increased at 1 week after the 2nd dose, were decreased 2 months later. Thus, the serum antibodies observed at early time points after the 2nd dose may be produced by plasmablasts. The spike RBD-specific memory B cells, which were increased after the 2nd dose, were further expanded after the 3rd dose. Studies of lymph node B cells have also shown that spike-specific germinal center B cells induced by two vaccine doses remained at high levels or even increased several months after immunization^63,64^. Thus, the enhanced antibody production after the 3rd dose seems to be due to the increase in RBD-specific memory B cells after the 2nd dose. These findings indicate that even though the serum titer decreases, spike RBD-specific memory B cells differentiate into antibody-secreting cells when exposed to SARS-CoV-2.

As mentioned in the introduction, there are conflicting reports regarding spike-specific T-cell responses elicited by the 3rd dose of the vaccine, and the frequency of spike-specific AIM^+^ T cells was not enhanced by the 3rd dose in our study. The reasons for the different findings among studies remain unknown, but there are some possible explanations for the low T cell responses to the booster vaccine. T cells expand upon antigen exposure, but the numbers of antigen-specific T cells decrease when the antigens are eliminated. Because memory T cells respond more rapidly, the spike-specific CD4^+^ T and CD8^+^ T cells observed at 2 months after the 3rd dose may be those T cells that remain after the contraction phases, and the frequencies of CD8^+^ T cells were not increased at this time point. In general, activated CD8^+^ T cells expand more rapidly than CD4^+^ T cells, and their contraction occurs more rapidly^65^. Goel et al. demonstrated that with two doses of the BNT162b2 mRNA vaccine, CD4^+^ T and CD8^+^ T cells contracted with half-lives of 47 and 28 days, respectively. In COVID-19 convalescent patients, memory T cell responses were biased toward CD4^+^ T cells^66^. While SARS-CoV-2 spike-specific CD4^+^ T cell responses were detected in all COVID-19 convalescent patients, CD8^+^ T cell responses against the spike were detected in 70% of these patients. Another possible scenario explaining low T cell responses to the booster vaccine would be that CD4^+^ T and CD8^+^ T cells expressing higher-affinity T cell receptors (TCRs) are selected after the 3rd dose. T cells with higher-affinity TCRs outcompete those with lower-affinity TCRs, and thus the vaccine booster may not increase the levels of antigen-specific T cells^65^. Indeed, mRNA vaccine-elicited antigen-specific T cell responses were not enhanced in individuals who had recovered from COVID-19 compared to SARS-CoV-2-naïve individuals, as shown by using AIM assays^52^. However, other studies have shown that whereas the cytokine-producing capacities of T cells elicited by two doses of the mRNA vaccine were reduced against the Omicron spike proteins, the reduction was not observed in individuals with prior SARS-CoV-2 infection or three-dose vaccinated individuals^43,51^. The studies that showed enhanced T cell reactivity after the 3rd dose were performed using cytokine producing assays^43,44^. Thus, both functional and numerical studies on spike-specific T cells are required to address the effect of booster vaccines on T cell memory.

Whereas repeated antigen exposure resulted in a decrease in T cell numbers by selecting T cells with high-affinity TCRs, B cells repeatedly activated by antigens generate new clones by producing new B cell receptors through somatic hypermutation. There are reports showing that the frequency of somatic hypermutation increased 6 months after SARS-CoV-2 mRNA vaccination^17,63^. Thus, the generation of B cells with different B cell receptors may contribute to the increase in spike RBD-specific B dcells by the vaccines. We observed a tendency for an increase in memory B cells specific to the Omicron spike protein at 6 months after the 2^nd^ dose. Other reports have shown that memory B cells that recognize spike proteins of variant strains are also gradually generated after the 2nd dose^60,62,67^. It is unknown how memory B cells continue to evolve even a few months after vaccination, but these findings suggest that there are already B cells that have the potential to respond to variant spikes even if there are no circulating antibodies reactive to those variants.

A study showed that coordinated SARS-CoV-2-specific adaptive immune responses of T cells and antibodies were associated with better protection against SARS-CoV-2^30^. In our study, although the kinetics of the mRNA vaccine-induced responses were different among circulating CD4^+^ T, CD8^+^ T and B cells, these adaptive immune responses appear to be orchestrated. The frequencies of S1-specific CD4^+^ T cells, cTfh cells and cTph cells at 1 week after the 2nd dose were positively correlated with those of spike RBD-specific IgG^+^ memory B cells after the 3rd dose. The positive correlation between the frequencies of AIM^+^ CD4^+^ T cells and CD8^+^ T cells at each time point was high. The frequencies of AIM^+^ CD4^+^ T cells at 1 week after the 2nd dose was also strongly correlated with those of AIM^+^ CD8^+^ T cells at all time points after the 2nd and 3rd doses, suggesting that the early CD4^+^ T cell response contributes to the induction of CD8^+^ T cells at later time points. While spike-specific CD4^+^ T cells, including Tfh cells, in the blood showed the peak response one week after the 2nd dose of BNT162b2, these T cells were maintained at high levels in the lymph nodes for at least 200 days^42^. The different kinetics of circulating CD4^+^ T cell responses and those in the lymphoid organs may explain the poor association of spike-specific CD4^+^ T cells with antibody responses after the 3rd dose, and early CD4^+^ T cell responsiveness induced by the two-dose vaccine may predict memory T and B cell responses in the long term.

Previously, we reported that age was negatively correlated with spike IgG levels in males, and this was associated with reduced cTph cell activation at 1 week after the 2nd dose^34^. The age-related reduction in the antibody response in males was also observed at all time points, but this reduction was milder after the 3rd dose. As the frequencies of cTph cells after the 2nd dose and memory B cells after the 3rd dose were positively correlated, individuals with weak CD4^+^ T cell responses at early time points may especially benefit from the 3rd dose. These findings emphasize the importance of the booster vaccine in individuals with weak CD4^+^ T cell responses to the primary vaccines.

The T cell responsiveness after the 3rd dose against the Omicron spike was 1.3-fold lower than that against the wild-type spike. The neutralizing capacity of serum against the Omicron spike protein after the 2nd dose was 17% that against the wild-type spike. However, the neutralizing capacity and frequency of memory B cells against the Omicron spike after the 3rd dose were 51% and 75% those against the wild-type spike, respectively. While T cells recognize peptide antigens, B cell epitopes can be continuous and discontinuous amino acid residues. This enables antibodies to recognize protein structure, but the binding of antibodies against such protein structures could be more easily impaired by mutation of the epitope. Thus, the booster dose may be more strongly required by B cells that are able to generate new B cell receptors.

There are limitations to this study. The neutralizing capacity of serum was evaluated by its capacity to inhibit the binding of SARS-CoV-2 spike RBD to the human angiotensin-converting enzyme 2 protein. However, to validate the capacity to inhibit viral infectivity, serum should be tested for neutralization activities against live SARS-CoV-2 or pseudoviruses bearing the SARS-CoV-2 spike protein. The limitations of this study include the sample size and status of the study participants. The sample size is small, with especially few individuals over 60 years old. We did not observe some associations among spike-reactive adaptive immune responses that have been reported by other groups, such as the association of IgG levels with memory B cells and CD4^+^ T cells^15,52,68,69^. Another caveat is that vaccination and blood collection were performed during the SARS-CoV-2 pandemic. We observed enhanced T cell reactivities against “N” among participants who were seronegative for anti-nucleocapsid antibody even though no participants were diagnosed with COVID-19 before or during this study. T cells that are cross-reactive with the SARS-CoV-2 nucleocapsid have been reported in individuals unexposed to the virus^66,70^. SARS-CoV-2 nucleocapsid-reactive T cells have also been observed in SARS-CoV-2 infection naïve individuals after vaccination^34,55,71^. Previously, we found that T cell reactivity against the nucleocapsid was positively correlated with T cell reactivity against CMV and influenza HA peptide pools, and thus, the enhanced T cell responses against unrelated antigens appeared to be due to bystander activation by the vaccine, which was also observed in vaccination with tetanus toxoid^72^. However, SARS-CoV-2 infection sometimes causes T cell responses without antibody responses. Thus, we are not able to exclude the possibility that some participants in the study were exposed to the virus.

We revealed that each component of the adaptive immune system responds differently to mRNA vaccines. The early CD4^+^ T cell responses elicited by the mRNA vaccine may predict the long-term immunological memory of B and T cells. Even after vaccine-induced antibody levels decreased, memory B cells, CD4^+^ T cells and CD8^+^ T cells were maintained for at least a few months. Because these memory cells exert effector functions and play crucial roles when they encounter the virus, cellular components should also be examined for future vaccine strategies.

## Methods

### Study design

A total of 71 participants, including 53 heath care workers who received three doses of the BNT162b2 (Pfizer/BioNTech) mRNA vaccine, were included in the study. All participants were Asian, and individuals who had been diagnosed with COVID-19 and/or were treated with systemic immunosuppressive agents were ineligible for the study. The characteristics of the study participants are summarized in Table 1. Peripheral blood samples were collected before, 1 week, and 2 and 6 months after the 2nd dose and 2 months after the 3rd dose. The timeline of vaccination and sample collection is presented in Fig. 1. This study was approved by the regional ethics committee at Juntendo University (M20-0253-M02 and M21-0285-M02) and conducted following the Declaration of Helsinki and Colombian legislation (Ministry of Health resolution 008430 de 1993). At enrollment, written informed consent was obtained from all participants.

**Table 1:** Characteristics of the study participants.

	T0	T1	T2	T3	B-T2
Total-n	71	71	68	68	71
HCW-n (%)	53 (75%)	53 (75%)	53 (78%)	53 (78%)	53 (75%)
**Sex-n (%)**					
Male-n (%)	41 (58%)	41 (58%)	39 (57%)	38 (56%)	41 (58%)
Female-n (%)	30 (42%)	30 (42%)	29 (43%)	30 (44%)	30 (42%)
**Age (years)**					
Median	36.0	36.0	35.0	35.0	36.0
IQR	(30.0–51.0)	(30.0–51.0)	(30.0–49.8)	(30.0–49.8)	(30.0–51.0)
Range	(20–79)	(20–79)	(20–79)	(20–79)	(20–79)
20–29-n (%)	17 (24%)	17 24%)	16 (24%)	16 (24%)	17 (24%)
30–39-n (%)	24 (34%)	24 (34%)	24 (35%)	24 (35%)	24 (34%)
40–49-n (%)	11 (15%)	11 (15%)	11 (16%)	11 (16%)	11 (15%)
50–59-n (%)	15 (21%)	15 (21%)	13 (19%)	13 (19%)	15 (21%)
≥ 60-n (%)	4 (6%)	4 (6%)	4 (6%)	4 (6%)	4 (6%)
**Days (median[IQR])**					
After 2nd dose	N/A	8 (7–9)	72 (68–76)	172 (168–178)	308 (282–315)
After 3rd dose	N/A	N/A	N/A	N/A	61 (57–64)

*HCW* health care worker, *IQR* interquartile range.

### PBMC and Serum Isolation

Fresh PBMCs were isolated from whole blood by using density-gradient centrifugation using a BD Vacutainer CP Mononuclear Cell Preparation Tube with Sodium Heparin (BD Biosciences, Franklin Lakes, NJ, USA). Fresh PBMCs were used for activation-induced marker assays and T and B cell Subset Analysis by Flow Cytometry, and the remaining PBMCs were cryopreserved in Bambanker (Nippon Genetics Co., Ltd., Tokyo, Japan) and stored in liquid nitrogen. Sera were separated from whole blood collected in tubes without anticoagulant, centrifuged at 1800 × g for 15 min and stored at − 80 °C.

### Activation-induced marker assay

One million fresh PBMCs were cultured in 96-well flat-bottom plates in TexMACS (Miltenyi Biotec, Bergisch Gladbach, Germany) containing the peptide pools (0.6 nmol/mL) at 37 °C in a 5% carbon dioxide incubator. The following PepTivator overlapping SARS-COV-2 peptide pools were obtained from Miltenyi Biotec: “S1” (the complete N-terminal S1 domain of the spike glycoprotein including RBD; aa 1–692), “S^+^” (the sequence domain aa 689–895 in the S2 domain of the spike glycoprotein), “S” (the immunodominant sequence domains of the spike glycoprotein), “N” (the complete sequence of the nucleocapsid phosphoprotein), a mutation pool of Prot_S B.1.1.529/BA.1, and a wild-type reference pool. A mixture of S1, S^+^, and S was used as the “S mix” peptide pool. A peptide pool of CMV pp65 protein were also obtained from Miltenyi Biotec. Each lyophilized peptide pool was reconstituted with sterile distilled water and used for AIM assays following the manufacturer’s instructions. Twenty-two hours later, the cells were incubated with Zombie Yellow Fixable Viability Dye and Fc receptor blocking solution (both from BioLegend, San Diego, CA, USA) and stained with a mixture of monoclonal antibodies against human cell-surface antigens in diluted form: anti-CD3-Brilliant Violet 421 (clone UCHT1), anti-CD4-APC/Fire 750 (clone RPA-T4), anti-CD8-PE (clone SK1), anti-OX40-FITC (clone Ber-ACT35), anti-CD137(4-1BB)-APC (clone 4B4-1), and anti-CD69-PE/Dazzle 594 (clone FN50) (all from BioLegend). Data were acquired on a FACS LSR Fortessa (BD Biosciences) and analyzed by using FlowJo software (TreeStar, Ashland, OR, USA).

### Measurement of anti-SARS-CoV-2 spike RBD IgG and anti-SARS-CoV-2 neutralizing antibodies

The serum concentration of anti-SARS-CoV-2 spike RBD IgG was measured by using an automated chemiluminescent microparticle immunoassay (Abbott Laboratories, Abbott Park, IL, USA). The serum neutralizing antibody levels for wild-type and Omicron spike proteins were measured by using an Anti-SARS-CoV-2 Neutralizing Antibody Titer Serologic Assay Kit and Anti-SARS-CoV-2 (B1.1.529) Neutralizing Antibody Titer Serologic Assay Kit (both from Acrobiosystems, Newark, DE, USA), respectively, according to the manufacturer’s instructions.

### T and B cell subset analysis by flow cytometry

PBMCs were incubated with Fc receptor blocking solution (BioLegend) and then stained with mixtures of the following monoclonal antibodies against human surface antigens: anti-CD3-Brilliant Violet 421 (clone UCHT1), anti-CD4-APC/Fire 750 (clone RPA-T4), anti-CD45RA-Brilliant Violet 605 (clone HI100), anti-CD279 (PD1)-PE (clone EH12.2H7), and anti-CD185 (CXCR5)-PE/Dazzle594 (clone J252D4) (all from Biolegend) for T cell subset analysis; anti-CD19-PE/Dazzle594 (clone HIB19), anti-CD20- Alexa Fluor 700 (clone 2H7), anti-CD27-APC/Fire 750 (clone M-T271), anti-IgD-Brilliant Violet 421 (clone IA6-2) (all from Biolegend), and anti-CD38-FITC (clone T16) (Beckman Coulter, Brea, CA, USA) for B cell subset analysis. Cells were washed and stained with 7-AAD Viability Staining Solution (BD Biosciences). Data were acquired on a FACS LSR Fortessa (BD Biosciences) and analyzed using FlowJo software (TreeStar Inc.).

### Detection of SARS-CoV-2 spike-specific B cells by flow cytometry

Recombinant biotinylated SARS-CoV-2 spike RBD proteins of the wild type and the Omicron variant (Acrobiosystems) were tetramerized with streptavidin APC and streptavidin BV605 (both from BioLegend) as previously described^17,52,57^. Two to four million cryopreserved PBMCs were first incubated with Fc receptor blocking solution and then stained with Fluorescent-labeled Spike RBD and mixtures of the following monoclonal antibodies against human surface antigens: anti-CD3- PerCP/Cyanine5.5 (clone UCHT1), anti-CD14-PerCP/Cyanine5.5 (clone M5E2), anti-CD20- Alexa Fluor 700 (clone 2H7), anti-CD27-APC/Fire 750 (clone M-T271), anti-IgM-PE/Cyanine7 (clone MHM-88), and anti-IgG Fc-Brilliant Violet 421 (clone M1310G05) (all from BioLegend); anti-CD8- PerCP/Cyanine5.5 (clone SK1), anti-CD19- V500 (clone HIB19), and anti-IgD-PE-CF594 (clone IA6-2) (all from BD Biosciences); and anti-CD38-FITC (clone T16) (Beckman Coulter). Cells were washed and stained with 7-AAD Viability Staining Solution (BD Biosciences). Data were acquired on a FACS LSR Fortessa (BD Biosciences) and analyzed using FlowJo software (TreeStar Inc.). Data at each time point were obtained by subtracting the frequency before the vaccine.

### Statistical analysis

Data were analyzed using GraphPad Prism 9.0 (GraphPad, La Jolla, CA, USA). Differences between groups were analyzed using appropriate tests as indicated in the figure legends. Associations between two variables were analyzed using Spearman’s correlation.

### Ethics approval

This study was conducted with the approval of the regional ethics committee at Juntendo University (M20-0253-M02 and M21-0285-M02). Informed consent was obtained from all patients and healthy volunteers.

## Supplementary Information

Supplementary Information.

## Data Availability

All data needed to evaluate the conclusions in the paper are presented in the article/Supplementary Material. Additional data and methods related to this paper may be requested from the authors.
