# Supplementary material for: Early CD4+ T cell responses induced by the BNT162b2 SARS-CoV-2 mRNA vaccine predict immunological memory
Source: Sci Rep. 2022 Nov 27;12:20376. doi: 10.1038/s41598-022-24938-4 (PMC9701808; doi:10.1038/s41598-022-24938-4)
Supplement: Supplementary file 1 — Supplementary Information. [file 41598_2022_24938_MOESM1_ESM.pdf]

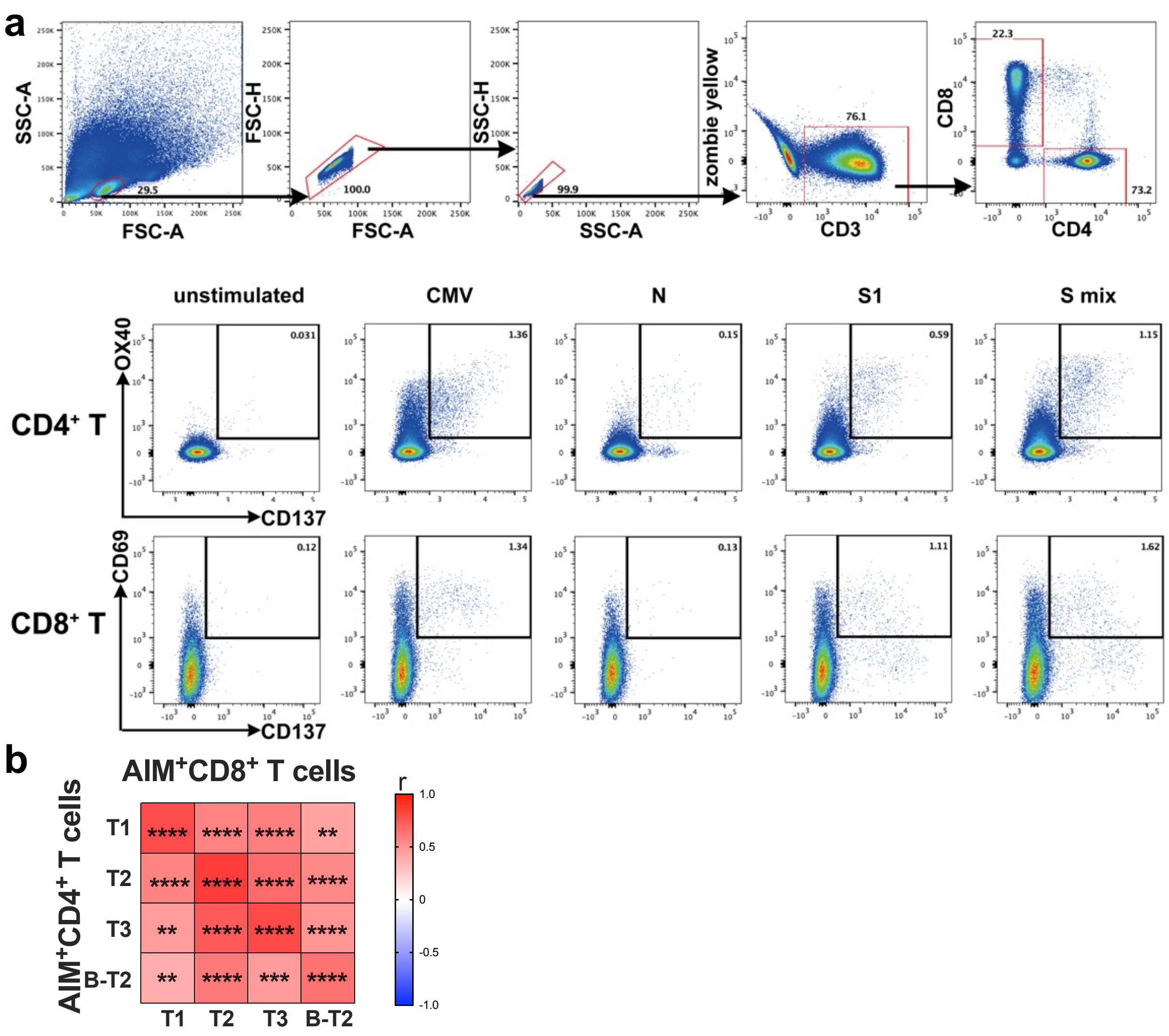

Supplementary Figure 1. Gating strategy used for AIM assays and correlations between SARS-CoV-2 mRNA vaccination-induced early CD4$^+$ T cell and booster vaccine-induced CD8$^+$ T cell responses.

(a) Lymphocytes were gated using a forward scatter area (FSC-A) vs. side scatter area (SSC-A) plot. Single cells were selected using FSC-A vs. FSC-height (FSC-H) and SSC-A vs. SSC-height (SSC-H) plots. Zombie yellow-positive dead cells were excluded, and CD4$^+$ and CD8$^+$ cells were gated among live CD3$^+$ cells. FACS plot examples of activation-induced marker (AIM)$^+$ (OX40$^+$CD137$^+$) CD4$^+$ T cells and AIM$^+$(CD69$^+$CD137$^+$) CD8$^+$ T cells are shown. (b) Heatmaps show the correlations among the frequencies of S1-reactive CD4$^+$ T and CD8$^+$ T cells at 1 week (T1), 2 months (T2), and 6 months (T3) after the 2nd dose of the BNT162b2 mRNA vaccine and 2 months after the 3rd dose (B-T2) from the experiments in Figure 1. Correlations were analyzed using Spearman's correlation analysis. $^{**}p < 0.01$, $^{***}p < 0.001$, $^{****}p < 0.0001$.

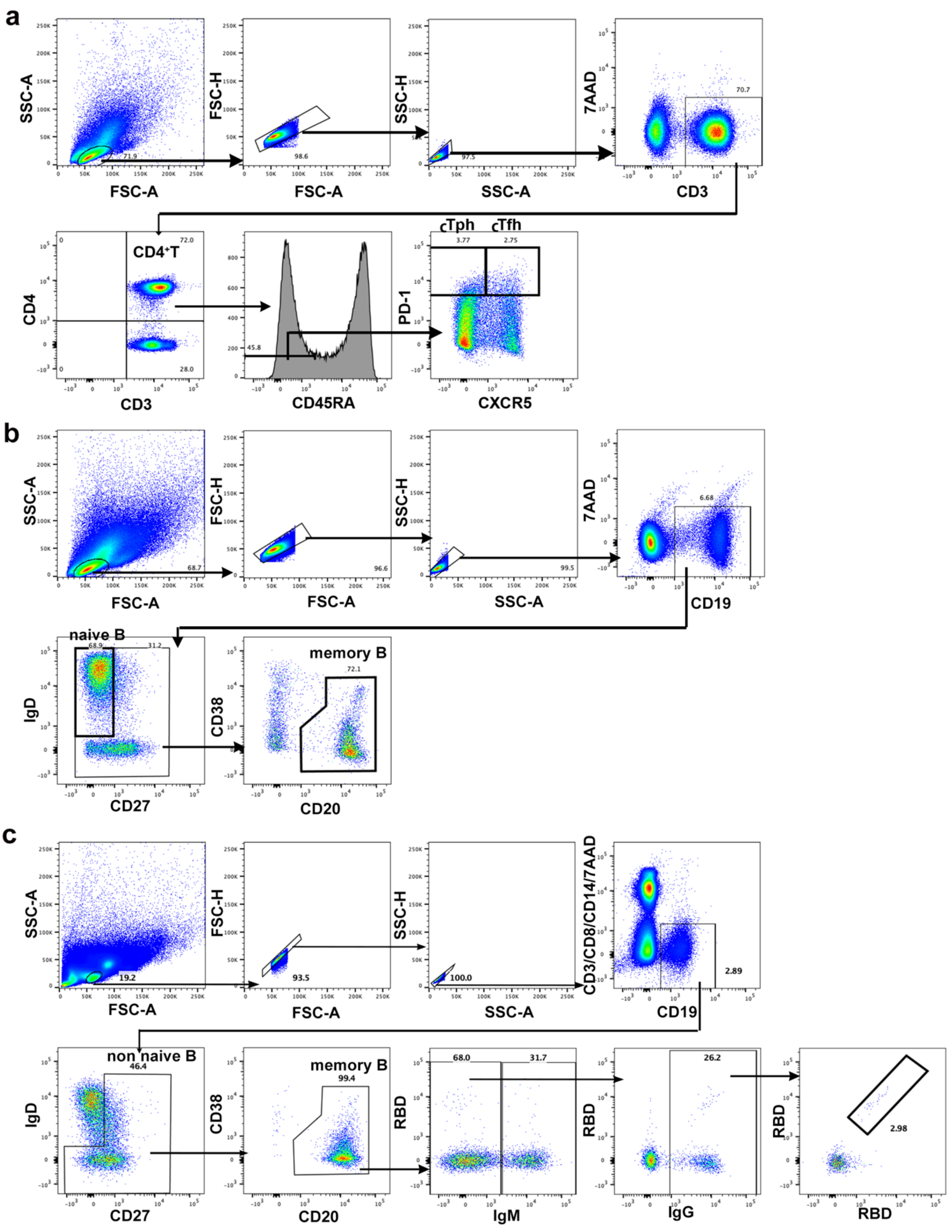

Supplementary Figure 2. Analysis of T and B cell subsets and spike-specific memory B cells induced by SARS-CoV-2 mRNA vaccines.

Gating strategies of circulating T follicular helper (cTfh) and circulating T peripheral helper (cTph) cells (a), naïve and memory B cells (b), and spike RBD-specific IgG^{+} memory B cells (c). (a-c) Lymphocytes were gated by using FSC-A vs. SSC-A plots, and single cells were selected by using FSC-A vs. FSC-H and SSC-A vs. SSC-H plots. 7-AAD-positive dead cells were excluded. (a) CD3^{+}CD4^{+}CD45RA^{-} cells were selected, and CXCR5^{+}PD1^{high} cTfh and CXCR5^{-}PD1^{high} cTph cells were gated as shown. (b) Among 7-AAD^{-}CD19^{+} cells, CD27^{-}IgD^{+} naïve cells and memory B cells are gated as shown. (c) CD19^{+} cells were

selected among 7-AAD$^-$CD3$^-$CD8$^-$CD14$^-$ cells, and CD27$^-$IgD$^+$ naïve B cells were excluded. Among nonnaïve B cells, memory B cells were selected based on the expression of CD20 and CD38. RBD-APC and RBD-BV-605 double-positive cells were gated among IgM$^-$IgG$^+$ memory B cells.

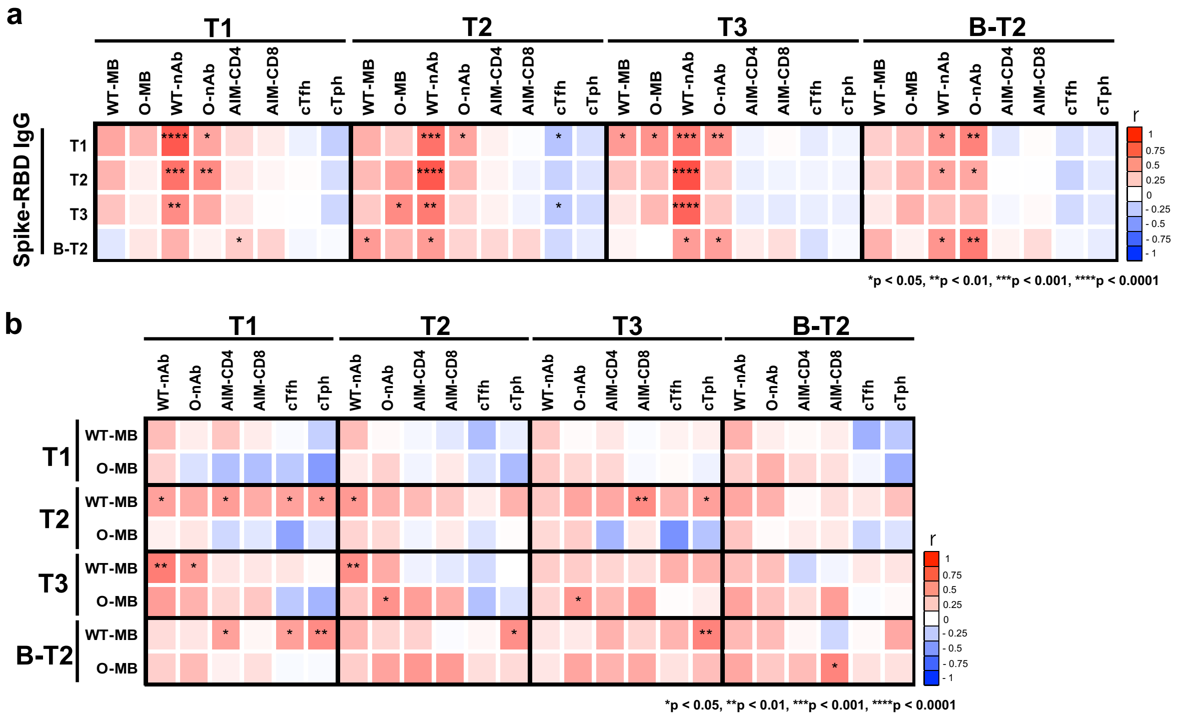

Supplementary Figure 3. Association of B cell responses with all parameters in the study.

Heatmaps show the correlations of serum spike RBD IgG levels with the indicated B cell and T cell frequencies at the indicated time points (a) and the correlations of the frequency of memory B cells specific for the RBD of wild-type (WT-MB) and omicron (O-MB), with the indicated antibody and T cell responses at the indicated time points (b). WT, wild-type spike; O, omicron spike; MB, memory B cells; nAb, neutralizing antibody titer; S1-CD4, S1-reactive $CD4^+$ T cells; S1-CD8, S1-reactive $CD8^+$ T cells; cTfh, circulating T follicular helper cells; cTph, circulating T peripheral helper cells. Correlations were analyzed using Spearman's correlation analysis. The Spearman correlation coefficient (r) is shown from red (1.0) to blue (-1.0). $*p < 0.05$, $**p < 0.01$, $***p < 0.001$, $****p < 0.0001$.
